# Sequential eradication of bacterial persisters: integrating phytochemical pharmacology with microenvironment-responsive delivery strategies

**DOI:** 10.3389/fmicb.2026.1842301

**Published:** 2026-05-26

**Authors:** Jixiang Bai, Lei Han, Xianzhi Cheng, Hengcai Fu, Xi Zhang, Huan He, Jia Wang

**Affiliations:** 1 Hongqi Hospital of Mudanjiang Medical University, Mudanjiang, China; 2 Harbin Hospital of Traditional Chinese Medicine, Harbin, China; 3 Xuyi County People's Hospital, Huai'an, Jiangsu, China; 4 Mudanjiang Medical University, Mudanjiang, China

**Keywords:** bacterial persisters, metabolic resuscitation, microenvironment-responsive delivery, plant-derived active monomers, sequential therapeutics

## Abstract

Bacterial persister cells within extracellular polymeric substance (EPS) matrices drive antimicrobial tolerance and chronic infection relapse. Conventional bactericidal agents remain fundamentally inadequate against these dormant subpopulations due to their reliance on active cellular metabolism. This review proposes a mechanistically driven, multi-phase sequential strategy—comprising barrier disruption, metabolic resuscitation, and terminal eradication—executed via highly purified, plant-derived natural products and advanced delivery systems. We synthesize recent pharmacological evidence regarding the anti-biofilm mechanisms of these active monomers and their integration with microenvironment-responsive strategies. A three-phase framework is delineated. Phase I utilizes epigallocatechin gallate (EGCG) and baicalin to physically degrade the EPS architecture and antagonize quorum sensing networks. Phase II employs Astragalus polysaccharides (APS) and exogenous metabolites to restore microbicidal host immunity and reactivate bacterial central carbon metabolism. Phase III leverages this reactivated state, utilizing berberine and shikonin to induce lethal reactive oxygen species (ROS) accumulation and terminal respiratory arrest. To resolve the pharmacokinetic limitations of these phytochemicals, we conceptualize integrating stimuli-responsive delivery systems for chronologically programmed drug release triggered by biofilm microenvironmental gradients. Ultimately, this sequential “disrupt-awaken-kill” strategy offers a potent framework to eradicate recalcitrant persisters, though translating these multi-component therapies into clinical practice requires overcoming existing manufacturing and regulatory complexities.

## 1 Introduction

The global antimicrobial resistance (AMR) crisis is exacerbated by bacterial persister cells residing within highly structured biofilms. Persisters represent a phenotypically tolerant, non-proliferative subpopulation of bacteria capable of surviving lethal antibiotic assaults without acquiring genetic resistance mutations (Fisher et al., 2017; Gollan et al., 2019). Concurrently, biofilms function as structural barriers encased in a self-produced EPS matrix, providing protection against both antimicrobial agents and host immune clearance mechanisms (Domouchtsidou et al., 2026). The clinical burden of this dual defense mechanism is substantial. According to the National Institutes of Health, biofilm formation is implicated in 65% of all microbial infections and approximately 80% of chronic and relapsing infections, including cystic fibrosis-associated pneumonia, diabetic foot ulcers, and medical device-related complications (Høiby et al., 2015; Sharma et al., 2023; Zafer et al., 2024). Eradicating these structured communities typically requires antibiotic concentrations 10 to 1,000 times higher than those effective against planktonic bacteria, necessitating prolonged, high-dose therapies that frequently result in treatment failure and severe host toxicity (Domouchtsidou et al., 2026; Hall and Mah, 2017).

Antibiotic persistence serves as a critical evolutionary precursor to full genetic resistance (Bakkeren et al., 2020; Van den Bergh et al., 2017). The recurrent clinical cycle of antibiotic-induced stress, persister survival, and post-treatment resuscitation creates a continuous reservoir of viable bacteria. Mathematical modeling and empirical evidence demonstrate that this persistence causes latent treatment failures, wherein surviving cells resume growth after antibiotic cessation (Witzany et al., 2022). This repeated cycle of killing and regrowth within the biofilm facilitates the accumulation of resistance mutations, thereby accelerating the global AMR crisis through sustained selective pressure (Bakkeren et al., 2020; Müsken et al., 2018).

The recalcitrance of persister cells exposes a fundamental limitation in conventional antibacterial pharmacotherapy. Bactericidal agents, including beta-lactams and fluoroquinolones, require active cellular machinery to exert lethal effects, specifically disrupting energy-dependent processes such as cell wall peptidoglycan construction and active DNA replication (Harms et al., 2016; Shan et al., 2017). Persisters evade these mechanisms by entering a state of metabolic dormancy and growth arrest, rendering antibiotic targets physiologically inactive or structurally inaccessible (Wainwright et al., 2021). This transition is regulated by the stringent response, a global transcriptional reprogramming governed by the accumulation of the alarmone (p)ppGpp via the RelA and SpoT enzymes (Harms et al., 2016; Svenningsen et al., 2019). In response to environmental stressors, elevated (p)ppGpp levels trigger the downregulation of ribosomal machinery, activate dormancy-inducing toxin-antitoxin (TA) systems, and upregulate generalized stress responses (Bryson et al., 2020; Patel et al., 2022).

**Figure 1:**
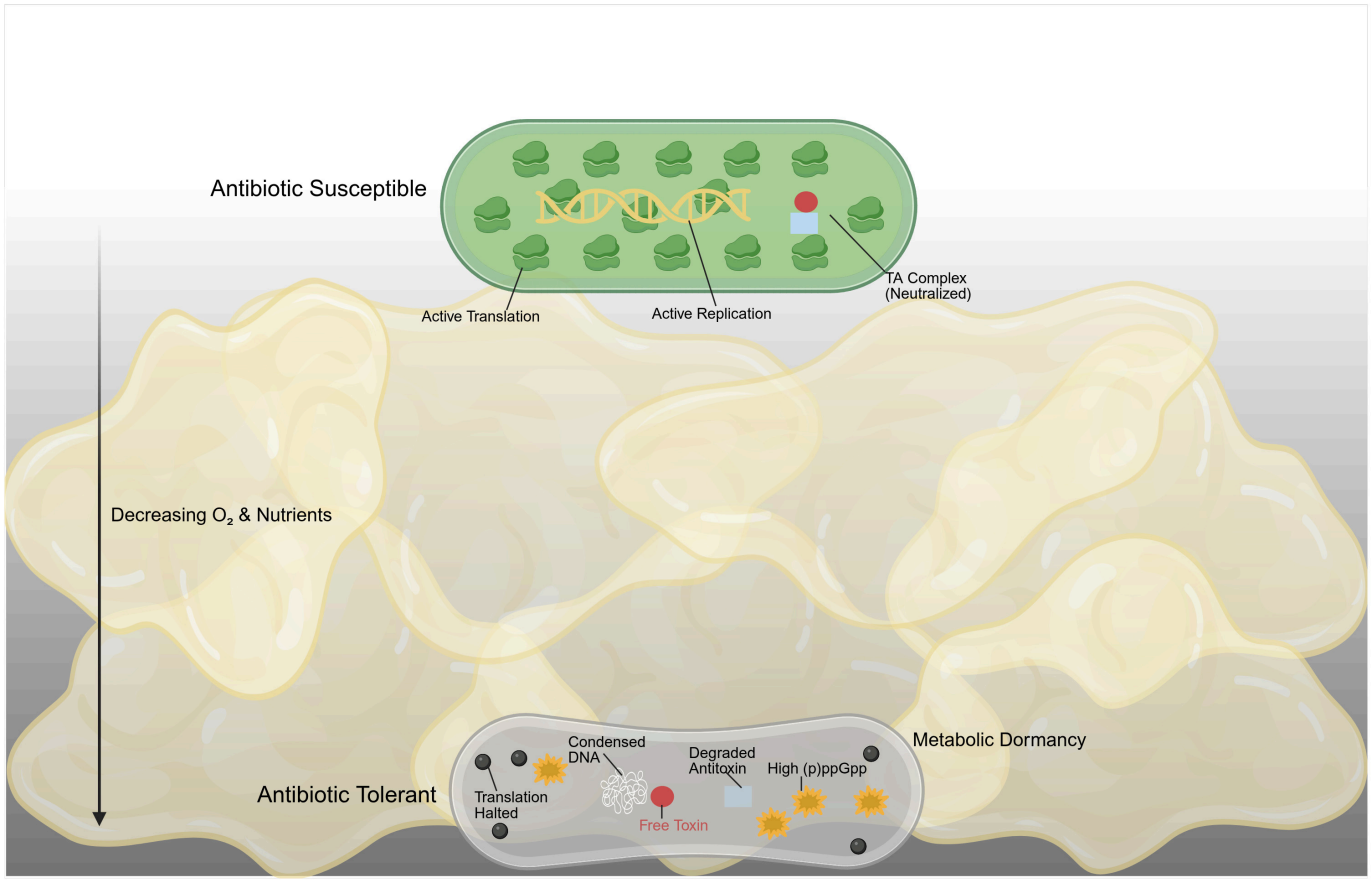
Spatial heterogeneity and physiological distinctness of bacterial persister cells within the biofilm architecture (created with BioRender.com). The schematic illustrates metabolic stratification driven by physicochemical gradients. **(Top)** Metabolically active bacteria in the nutrient- and oxygen-rich periphery exhibit active translation and DNA replication, rendering them susceptible to bactericidal antibiotics. **(Bottom)** Persister cells residing in nutrient-deprived deep layers adopt a dormant phenotype, characterized by condensed nucleoids, halted translation, and ATP depletion. This profound dormancy and subsequent antibiotic tolerance are molecularly driven by the activation of toxin-antitoxin (TA) systems and the stringent response, mediated by the accumulation of the (p)ppGpp alarmone.

The EPS matrix provides secondary physical and chemical protection. This matrix acts as a diffusion barrier, impeding antibiotic penetration while sequestering drugs through electrostatic binding to negatively charged extracellular DNA (eDNA) and matrix proteins (Böhning et al., 2024; Pinto et al., 2020). Furthermore, biofilms exhibit pronounced spatial heterogeneity; oxygen and nutrient limitations in deeper biofilm layers establish steep chemical gradients. These microenvironmental stressors actively induce the stringent response and force deeply embedded cells into dormant states prior to antibiotic exposure (Stewart et al., 2019). The interaction between quorum sensing (QS) communications, biofilm formation, and phenotypic tolerance creates a synergistic mechanism that effectively limits the efficacy of conventional monotherapies (Sionov and Steinberg, 2022). The interplay of these microenvironmental stressors, elevated (p)ppGpp levels, and the resulting phenotypic transition into a dormant state is illustrated in Figure 1.

To address these multifaceted tolerance mechanisms, contemporary therapeutic paradigms are shifting from traditional single-target antibiotics toward staged, multi-target sequential therapies (Koo et al., 2017; Li et al., 2026). This strategy systematically dismantles biofilm defenses through a defined sequence. Initially, the EPS matrix must be disrupted to eliminate physical barriers and quench QS networks (Ruiz-Sorribas et al., 2022). Subsequently, dormant persisters must be metabolically resuscitated. Exogenous metabolites, including specific sugars and amino acids, transiently stimulate bacterial respiration and restore the proton motive force (PMF) independently of full growth resumption (Allison et al., 2011; Kitzenberg et al., 2022). This metabolic reprogramming reopens antibiotic targets and facilitates the active cellular uptake of bactericidal agents, rendering previously refractory populations highly susceptible to terminal bactericidal interventions (Batchelder et al., 2024; Song et al., 2026).

**Figure 2:**
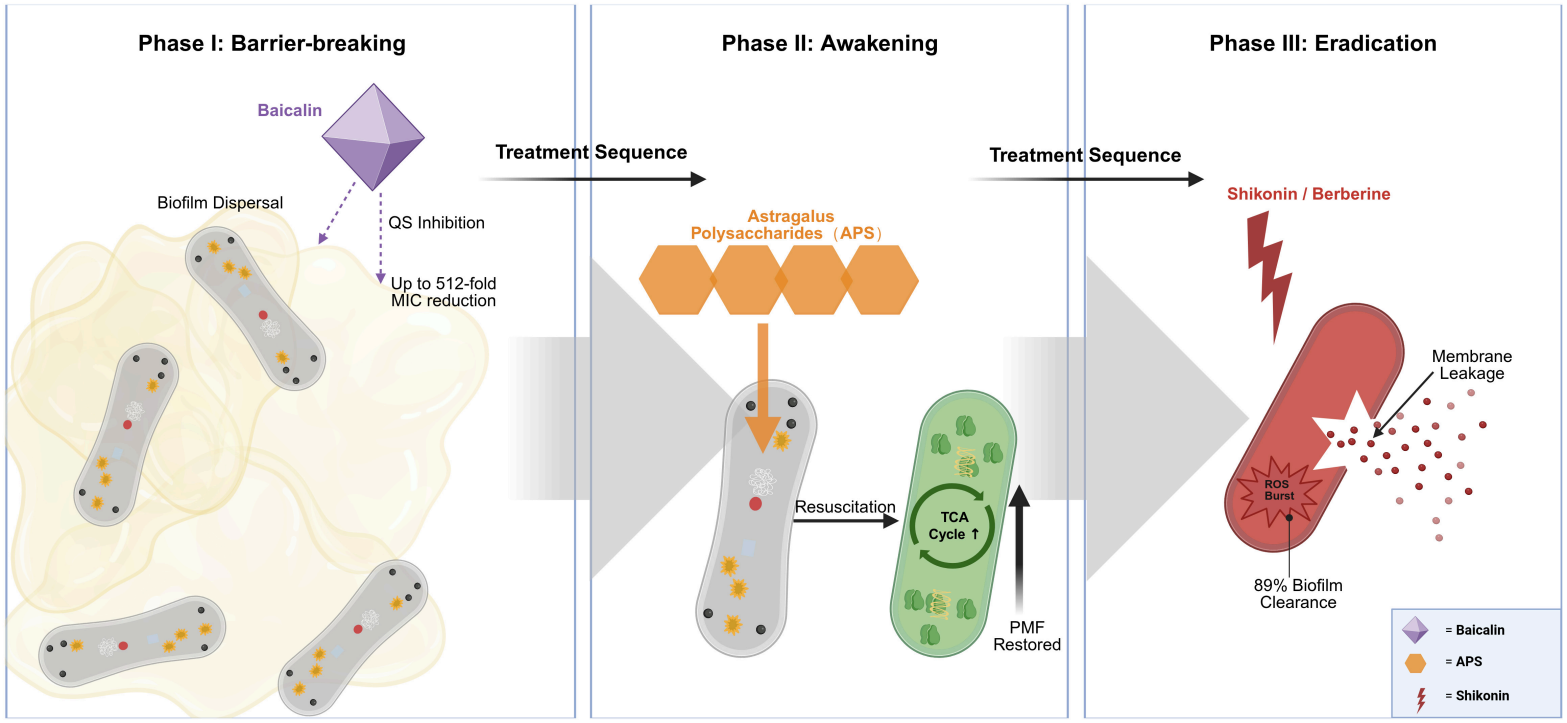
The sequential “Disrupt–Awaken–Kill” therapeutic paradigm for the eradication of biofilm-associated persisters (created with BioRender.com). The schematic delineates a three-phase intervention utilizing TCM-derived natural products. Phase I (barrier disruption): application of quorum sensing (QS) inhibitors, such as baicalin, to dismantle the EPS matrix and disperse aggregated persister cells. Phase II (metabolic resuscitation): exogenous metabolic primers (e.g., Astragalus polysaccharides) stimulate tricarboxylic acid (TCA) cycle flux and regenerate the proton motive force (PMF), transitioning dormant cells into a physiologically active state. Phase III (terminal eradication): administration of bactericidal agents, including shikonin or berberine. These compounds exploit the reactivated metabolic pathways to induce lethal reactive oxygen species (ROS) bursts and severe membrane permeabilization, culminating in cellular eradication.

Translating multi-agent sequential regimens into clinical practice is hindered by pharmacokinetic incompatibilities and the challenge of coordinating precise temporal delivery. TCM presents a unique translational potential in this context. Within the paradigm of modern pharmacology, TCM functions as an extensive reservoir of structurally diverse, highly purified active monomers and secondary metabolites. Characterized by a multi-component, multi-target pharmacological profile, these TCM-derived natural products possess the molecular diversity required to execute this complex sequential strategy (Luo et al., 2020). In this perspective, we delineate how rationally selected TCM-derived phytochemicals can sequentially execute an EPS disruption, metabolic resuscitation, and terminal bactericidal strategy. By integrating synergistic phytochemical properties with established microbiological mechanisms, we propose a comprehensive pathway to dismantle biofilm architecture, reverse metabolic dormancy, and eradicate recalcitrant persister infections. The overarching conceptual framework of this sequential intervention—comprising barrier disruption, metabolic resuscitation, and terminal eradication—is depicted in Figure 2.

## 2 EGCG-mediated structural matrix remodeling and chemical quenching

Epigallocatechin gallate (EGCG), a primary polyphenolic compound, executes a validated dual-targeted intervention: irreversible physical interference with EPS macromolecular assembly and highly specific antagonism of QS signaling cascades (Cheng et al., 2024; Serra et al., 2016).

Physically, EGCG compromises the structural resilience of the biofilm by directly remodeling functional amyloid fibers, which serve as the primary mechanical and adhesive scaffold for the EPS matrix across diverse bacterial taxa. In *Escherichia coli*, EGCG exerts a multi-tiered inhibitory effect on curli fiber biogenesis. At the post-translational level, EGCG physically impairs the polymerization of the major and minor curli subunits, CsgA and CsgB, into mature amyloid fibrils. Concurrently, at the transcriptional level, EGCG triggers the σE-dependent cell envelope stress response. This specific stress signaling activates the small regulatory RNA RybB, which directly targets and degrades csgD mRNA. The subsequent downregulation of the master biofilm regulator CsgD halts the biosynthesis of both curli and cellulose (Serra et al., 2016). Furthermore, EGCG accelerates the degradation of the stationary-phase sigma factor RpoS via the adenosine triphosphate (ATP)-dependent ClpXP protease complex, effectively eliminating the upstream transcriptional activation required for curli expression (Arita-Morioka et al., 2018). In Pseudomonas aeruginosa, EGCG interacts directly with the highly amyloidogenic sequences and linker regions of functional amyloid (Fap) proteins. This physiochemical interaction redirects Fap monomers away from their normal assembly pathway, forcing them into off-pathway, non-amyloid oligomeric aggregates (Najarzadeh et al., 2019; Stenvang et al., 2016). Quantitative nanoindentation analyses demonstrate that this specific amyloid remodeling significantly reduces the macroscopic mechanical stiffness of the biofilm matrix, thereby increasing its permeability to subsequent pharmacological agents (Stenvang et al., 2016). Beyond amyloid fibers, EGCG directly inhibits the transcription of the mdoH gene, which encodes crucial enzymes for polysaccharide biosynthesis, resulting in a substantial reduction of total EPS biomass (Zhang et al., 2023).

Chemically, EGCG functions as a potent, broad-spectrum QS inhibitor. Crucially, it exerts this anti-QS activity at sub-inhibitory concentrations (25–150 μg/mL), which neutralizes bacterial virulence without affecting cellular viability, thereby minimizing the selective pressure that drives genetic resistance (Sikdar et al., 2026). Comprehensive transcriptomic profiling and molecular docking simulations reveal that EGCG directly binds to the ligand-binding domains of multiple QS receptor proteins, including CviR, LasR, and RhlR. This competitive binding strictly obstructs the interaction between these receptors and their cognate autoinducers (Kim et al., 2019; Sikdar et al., 2026). By downregulating the synthesis of specific signaling molecules, namely *N*-acyl homoserine lactones (AHLs) and autoinducer-2 (AI-2), EGCG suppresses the entire downstream transcription of virulence regulons. This suppression precipitously reduces the secretion of critical biofilm-associated protective factors, including pyocyanin, tissue-degrading elastase, and surfactant rhamnolipids (Cheng et al., 2024; Tang et al., 2022). This combined degradation of structural scaffolding and silencing of chemical communication fundamentally dismantles the microenvironment required to maintain the persister phenotype.

## 3 Flavonoid-mediated quorum sensing inhibition and matrix degradation

Baicalin, a predominant flavonoid extracted from *Scutellaria baicalensis*, executes a comprehensive suppression of interconnected QS circuits while simultaneously halting EPS biosynthesis. In Pseudomonas aeruginosa, baicalin administered at sub-inhibitory concentrations (16–64 μg/mL) dose-dependently represses the transcription of the Las, Rhl, and Pseudomonas quinolone signal (PQS) regulatory genes (Luo et al., 2017). A primary mechanism driving this broad suppression is the direct functional inhibition of energy-dependent drug efflux pumps, specifically MexAB-OprM and MsrA. By disrupting these transmembrane transport systems, baicalin drastically restricts the extracellular export of autoinducers, including 3-oxo-C12-HSL and C4-HSL (Wang et al., 2019; Wang W. et al., 2023). This restriction prevents the threshold extracellular accumulation of signaling molecules necessary for receptor activation, thereby starving the QS network. In *Staphylococcus aureus*, baicalin explicitly targets the accessory gene regulator (Agr) QS system by significantly reducing the transcription of agrA, RNAIII, and sarA (Wang et al., 2019). This suppression correlates with reduced ATP content and diminished pyruvate kinase activity, confirming direct interference with energy-dependent efflux systems. Furthermore, baicalin interrupts AI-2-mediated interspecies communication. Molecular docking validates that baicalin binds with high affinity (binding energy of −6.65 kcal/mol) to the AI-2 transporter protein LsrB, effectively blocking signal perception, recognition, and subsequent cellular uptake (Li et al., 2025).

Parallel to its potent QS inhibitory activity, baicalin actively degrades the physical barriers of the biofilm matrix by transcriptionally downregulating genes encoding critical structural components. In *S. aureus* and *Staphylococcus saprophyticus*, baicalin specifically targets the icaADBC operon, which is essential for polysaccharide intercellular adhesin (PIA) or poly-*N*-acetylglucosamine (PNAG) biosynthesis (Chen et al., 2016; Peng et al., 2019). In *Escherichia coli*, baicalin downregulates curli biosynthesis genes (csgA and csgB) alongside the stationary-phase sigma factor rpoS (Peng et al., 2019). This dual inhibition of proteinaceous fibers and exopolysaccharides prevents early-stage bacterial adhesion and microcolony aggregation (Peng et al., 2019; Wang et al., 2021). Additionally, baicalin inhibits bacterial cell autolysis, thereby reducing the release of eDNA that normally contributes to matrix stability. Confocal laser scanning microscopy (CLSM) and field emission scanning electron microscopy (FE-SEM) confirm that baicalin treatment induces the loss of characteristic three-dimensional biofilm architecture, resulting in significantly decreased biofilm thickness and biomass. By loosening this EPS scaffold, baicalin fundamentally alters biofilm topography and enhances the penetrability of conventional antimicrobials. Combination therapies utilizing baicalin alongside tobramycin, ciprofloxacin, or gentamicin yield synergistic bacterial clearance rates in murine peritoneal implant and cutaneous infection models (Luo et al., 2017; Slachmuylders et al., 2018).

Other plant-derived secondary metabolites execute parallel anti-biofilm mechanisms, establishing a broader pharmacological class effect. The isoquinoline alkaloid berberine downregulates QS genes (luxS, qseB, qseC) and motility-associated genes (motA, fliC) in antimicrobial-resistant *E. coli* (Sun et al., 2019). The phenolic compound paeonol attenuates QS-regulated virulence in P. aeruginosa by repressing the lasI/R and rhlI/R systems, alongside downstream virulence factors such as elastase and pyocyanin (Yang et al., 2021). The flavonoid isoliquiritin demonstrates high-affinity binding (binding energy of −8.4 kcal/mol) directly to the RhlI active site via specific amino acid interactions (Trp34, Asp35, Tyr105), substantially inhibiting biofilm formation in metallo-β-lactamase-producing strains (Fakhar et al., 2024). The antibiofilm efficacy of these compounds is strictly governed by defined structure-activity relationships. The precise positioning of multiple hydroxyl groups on aromatic rings enhances binding affinity to QS receptor proteins and bacterial enzymes. Furthermore, specific glycosylation patterns (such as those observed in baicalein-7-*O*-glucuronide) and moderate lipophilicity facilitate optimal membrane penetration while maintaining the aqueous solubility required for robust interaction within the EPS matrix (Gon Alves et al., 2023; Silva et al., 2023).

## 4 Phase II: Microenvironment remodeling and metabolic resuscitation

### 4.1 The interdependent host-pathogen tolerance axis

Physical disruption of the biofilm matrix is insufficient for comprehensive bacterial eradication. The host immune microenvironment and the bacterial metabolic state operate as a bidirectionally regulated, interdependent tolerance system. During the innate immune oxidative burst, host-derived ROS and reactive nitrogen species (RNS) directly induce bacterial metabolic dormancy (Beam et al., 2021). Specifically, macrophage-produced peroxynitrite (ONOO^−^) mediates antibiotic tolerance during phagocytosis. This host-driven physiological interaction supersedes the intrinsic bacterial persistence mechanisms observed in standard *in vitro* cultures (Beam et al., 2021). Furthermore, host cell-induced oxidative stress causes severe depletion of bacterial ATP. This energy deficit triggers the recruitment of the DnaK-ClpB chaperone system, initiates widespread protein aggregation, and drives intracellular *Staphylococcus aureus* persisters into a reversible dormant state. Critically, these dormant persisters retain their infectious capacity upon exit from the high-oxidative-stress microenvironment (Peyrusson et al., 2022).

Concurrently, intact biofilm architectures actively suppress localized host immune functions. *S. aureus* biofilms attenuate macrophage phagocytosis and drive immune responses toward an anti-inflammatory M2 macrophage polarization. This shift is characterized by the pronounced downregulation of microbicidal inducible nitric oxide synthase (iNOS) and the concurrent induction of arginase-1 (Thurlow et al., 2011). Biofilm-conditioned media upregulate the expression of Kruppel-like factor 2 (KLF2) in host macrophages. Elevated KLF2 attenuates nuclear factor-kappa B (NF-κB) signaling, thereby suppressing bactericidal activity and pro-inflammatory cytokine production (Alboslemy et al., 2019). Additionally, a localized CXCL16/CXCR6/TGF-β feedback loop between myeloid-derived suppressor cells (M-MDSCs) and regulatory T cells (Tregs) severely inhibits antibacterial immunity at the infection site (Wu X. et al., 2025). Therefore, effective persister eradication strictly requires a dual-targeted intervention: restoring pro-inflammatory microbicidal immune function to clear dispersed bacteria, alongside directed bacterial metabolic resuscitation to restore pharmacological susceptibility (Hanke et al., 2013; Lu et al., 2025).

### 4.2 APS-mediated immunomodulation and microecological restoration

APS execute a sophisticated dual-role intervention within the infectious microenvironment: driving host-directed immune restoration as intact macromolecules, while providing localized metabolic primers upon targeted enzymatic degradation. As a prebiotic substrate, intact APS selectively enriches short-chain fatty acid (SCFA)-producing gut microbiota taxa, specifically increasing the relative abundance of *Dubosiella, Lactobacillus*, and *Bifidobacterium* (Huo et al., 2024; Zhang et al., 2025a). Fecal microbiota transplantation (FMT) models confirm that the therapeutic efficacy of APS is strictly microbiota-dependent, as pharmacological benefits are entirely abolished in antibiotic-depleted murine models (Zhang et al., 2025a). Metatranscriptomic analyses indicate that microbial fermentation of APS significantly upregulates carbohydrate-active enzymes (CAZymes) and butyrate production genes (Rong et al., 2025). Butyrate, the primary SCFA elevated by APS fermentation, functions as an epigenetic regulator by directly inhibiting histone deacetylase 3 (HDAC3) in macrophages (Schulthess et al., 2019). This specific HDAC3 inhibition programs monocyte-to-macrophage differentiation toward a highly microbicidal phenotype. This reprogrammed phenotype exhibits enhanced phagocytosis, increased LC3-associated autophagy for intracellular bacterial degradation, and stabilized hypoxia-inducible factor 1α (HIF-1α) expression. Consequently, macrophages elevate the production of antibacterial effectors, including the cathelicidin antimicrobial peptide LL-37 in respiratory epithelial cells, without inducing an excessive or tissue-damaging inflammatory cytokine release (Liu et al., 2023; Schulthess et al., 2019; Zhao et al., 2018).

Independent of prebiotic microbiome modulation, APS directly activates innate immune cells through Toll-like receptor 4 (TLR4)-mediated, MyD88-dependent signaling pathways. This activation cascades through TRAF-6 to activate NF-κB and AP-1, stimulating the controlled release of essential cytokines (TNF-α, IL-1β, IL-6) (Wang et al., 2025; Zhou et al., 2017). This dual regulatory mechanism facilitates context-dependent macrophage polarization. Within highly immunosuppressive biofilm microenvironments, APS promotes M1 macrophage polarization, thereby increasing localized ROS generation and enhancing phagocytic capacity (Li L. et al., 2022; Zhang et al., 2025b). Conversely, in systemic hyperinflammatory states such as sepsis, APS and its bioactive component astragaloside IV restrict excessive M1 polarization and inhibit the formation of neutrophil extracellular traps (NETs). This is achieved via direct binding to IκBα, which attenuates overactive NF-κB signaling and prevents collateral endothelial tissue damage (Wu S. et al., 2025; Yang et al., 2024). Furthermore, APS induces dendritic cell (DC) morphological maturation and upregulates surface activation markers (CD80, CD86). This DC activation elicits robust T cell proliferation, shifting the immune response toward a Th1 profile and strengthening long-term adaptive immune surveillance against persistent bacterial pathogens (An et al., 2022; Liu et al., 2011).

### 4.3 Pathogen-directed metabolic resuscitation via polysaccharide degradation

To eliminate residual bacteria that evade immune clearance, dormant persister cells must be metabolically resuscitated to restore susceptibility to bactericidal antibiotics. Within the complex, enzyme-rich biofilm microenvironment, macromolecules such as APS are subjected to localized hydrolysis. Recent structural analyses confirm that bacterial carbohydrate-active enzymes, including β-galactosidases and endo-α-1,4-glucanases, efficiently degrade the α-1,4-linked glucose backbones of APS, releasing specific constituent monosaccharides, primarily free glucose (Cai et al., 2023; Li K. et al., 2022). These localized polysaccharide breakdown products act as potent metabolic primers. Empirical evidence utilizing ^13^C-isotope tracing demonstrates that polysaccharide-derived glucose is actively transported into bacterial cells, fueling the Embden–Meyerhof–Parnas (glycolysis) pathway and accelerating flux through the tricarboxylic acid (TCA) cycle (Xiong et al., 2018).

Crucially, simpler carbon sources like glucose are recognized as the most ubiquitous and efficient substrates for resuscitating persister variants across diverse bacterial species (Orman and Brynildsen, 2013; Sun et al., 2025). Exogenous glucose entry effectively resuscitates starvation-induced dormancy by upregulating gene networks associated with cellular repair and central metabolism (Kim et al., 2023; Prax et al., 2016). Concurrently, this glycolytic conversion to pyruvate generates increased concentrations of nicotinamide adenine dinucleotide (NADH) (Peng et al., 2015). The generated NADH transfers electrons to Complex I (NADH dehydrogenase) of the electron transport chain (ETC), driving transmembrane proton pumping and regenerating the PMF (Peng et al., 2015). The regeneration of this electrochemical gradient is strictly required to drive the active intracellular uptake of aminoglycosides and other bactericidal antibiotics, functioning entirely independent of cellular replication (Allison et al., 2011).

Alternatively, C4-dicarboxylates, including fumarate and succinate, completely bypass the glycolytic pathway. Fumarate is imported via transporters regulated by the alternative sigma factor RpoN (σ54) and enters the TCA cycle directly via fumarase, rapidly accelerating metabolic flux and ETC activity (Crabbé et al., 2019; Hall et al., 2019). Glycerol metabolism, mediated by glycerol kinase (GlpK), provides exceptionally rapid central metabolism integration and PMF enhancement. This specific pathway facilitates the complete eradication of methicillin-resistant *S. aureus* (MRSA) persisters within minutes of initial co-treatment (Xing et al., 2025). Pharmacological inhibition of the ETC utilizing sodium azide (targeting cytochrome c oxidase) or protonophores (e.g., CCCP) completely abolishes metabolite-induced aminoglycoside potentiation, definitively confirming that PMF regeneration is the essential prerequisite for bactericidal efficacy (Chen et al., 2019). Alternative metabolic activation pathways provide additional eradication targets. Exogenous thymine supplementation upregulates TCA cycle activity and stimulates global cellular respiration. This activation promotes the concurrent production of ATP and endogenous ROS, effectively sensitizing Gram-negative pathogens to DNA-damaging agents (ciprofloxacin) and cell wall inhibitors (ampicillin) in a strictly growth phase-independent manner (Liu et al., 2021). Furthermore, fatty acid signaling molecules, such as *cis*-2-decenoic acid (*cis*-DA), modify the transcriptional profile of key metabolic markers, including acyl carrier proteins and ATP synthase subunits. This targeted transcriptional shift increases basal respiratory activity and reverts dormant persisters to a highly antimicrobial-susceptible physiological state (Marques et al., 2014; Wang et al., 2018).

### 4.4 Synergistic dual-targeting interventions

The pharmacological reprogramming of host cell metabolism serves as a potent strategy to further reduce intracellular bacterial drug tolerance. Shifting macrophage metabolism from oxidative phosphorylation (OXPHOS) to glycolysis—achieved via the administration of the FDA-approved compound meclizine—generates significant mitochondrial ROS through reverse electron transport. This metabolic shift imposes severe intracellular oxidative stress on reductive mycobacterium tuberculosis persisters, substantially enhancing the bactericidal efficacy of frontline therapeutics (Yadav et al., 2025). Concurrently, host-directed adjuvants, such as KL1, directly modulate host immune response genes to suppress the excessive production of reactive species within macrophages. By strategically alleviating specific ROS and RNS stressors that act as primary triggers for dormancy, KL1 successfully sensitizes intracellular persister populations to antibiotics without promoting dangerous bacterial outgrowth (Lu et al., 2025).

Plant-derived small molecules provide critical adjunctive, metabolism-dependent bactericidal mechanisms. Bakuchiol, a natural phenolic compound, selectively disrupts bacterial membrane phospholipids. In *A. baumannii* persisters, bakuchiol damages localized phospholipid patches within the outer membrane, facilitating severe membrane permeabilization. This mechanism exhibits profound synergy with colistin, resulting in the mutual reinforcement of bactericidal effects (Suh et al., 2025). Exogenous organic acids, including malic acid and sodium acetate, significantly potentiate the efficacy of antibiotics against mature *P. aeruginosa* biofilms. These compounds function by actively increasing the bacterial intracellular pH, elevating endogenous ROS production, and accelerating TCA cycle activity to overcome tolerance (Bao et al., 2022). Finally, directly targeting bacterial bioenergetics with promethazine alters the transmembrane potential and interferes with cellular respiration. By preventing biofilm-grown bacteria from counteracting intracellular alkalization, promethazine completely abolishes biofilm-associated drug tolerance, effectively sensitizing deeply embedded, metabolically recalcitrant cells to multiple structural classes of antibiotics (Donnert et al., 2020).

## 5 Phase III: Terminal bactericidal killing mechanisms

Metabolic resuscitation transitions dormant persister cells into a physiologically active state, restoring susceptibility to bactericidal interventions. This terminal phase utilizes specific plant-derived secondary metabolites, primarily the alkaloid berberine and the naphthoquinone shikonin, as the definitive killing agents. These compounds exploit the newly reactivated metabolic pathways—specifically TCA cycle flux, electron transport chain (ETC) activity, and active macromolecular synthesis—to generate irreversible cytotoxic damage (Du et al., 2020; Liu et al., 2024; Watson et al., 2023; Yang et al., 2025).

### 5.1 Berberine-mediated multi-target lethality

Berberine induces terminal bactericidal effects through the severe perturbation of carbohydrate metabolism and oxidative phosphorylation. Quantitative proteomic analyses demonstrate that berberine exposure results in the lethal intracellular accumulation of ROS. The causative role of this oxidative stress is confirmed by the application of the antioxidant *N*-acetyl-l-cysteine, which completely abolishes berberine-induced antibacterial effects (Du et al., 2020). Furthermore, metabolomic profiling reveals that berberine compromises bacterial antioxidant defenses by depleting farnesyl pyrophosphate (the synthetic precursor to the antioxidant pigment staphyloxanthin) and reducing intracellular γ-tocopherol levels. This depletion corresponds with a significant accumulation of oxidized phospholipids within the bacterial membrane (Wu et al., 2022).

Beyond ROS generation, berberine structurally compromises bacterial membrane integrity. The quaternary ammonium structure of berberine facilitates direct electrostatic interaction with negatively charged membrane phospholipids. This interaction increases membrane permeability, disrupts the electrochemical gradient, and severely depletes intracellular ATP concentrations (Liu et al., 2024; Yang et al., 2025). Additionally, berberine directly inhibits bacterial cell division by targeting the essential GTPase FtsZ. Saturation transfer difference nuclear magnetic resonance (NMR) spectroscopy indicates that the dimethoxy groups and isoquinoline nucleus of berberine bind with high affinity to a hydrophobic pocket on FtsZ. This binding destabilizes FtsZ protofilaments and disrupts Z-ring midcell localization (Domadia et al., 2008). Genetic validation demonstrates that RNA silencing of ftsZ significantly sensitizes bacteria to berberine treatment, whereas ftsZ overexpression provides measurable rescue (Boberek et al., 2010). Berberine also acts as a DNA gyrase inhibitor, interfering with essential nucleic acid synthesis in ESKAPE pathogens (Saha et al., 2026).

### 5.2 Naphthoquinone-driven respiratory arrest and ATP depletion

Shikonin, a naphthoquinone derivative, directly targets and inhibits bacterial F_0_F_1_-ATP synthase, the terminal enzyme required for oxidative phosphorylation. Enzymatic assays confirm that shikonin achieves complete inhibition of wild-type ATP synthase. Mutational analyses identify specific residues (αR283D, αE284R, βV265Q, and γT273A) as essential components of the phytochemical binding site; structural alterations to these residues eliminate shikonin binding and mitigate growth inhibition (Watson et al., 2023). Additionally, shikonin exhibits high specific affinity for bacterial peptidoglycan. When administered concurrently with membrane-permeabilizing agents or ATPase inhibitors, the bactericidal activity of shikonin is potentiated by up to 75%, indicating the direct involvement of transmembrane ABC transporters (Lee et al., 2015).

The general bactericidal mechanism of the 1,4-naphthoquinone class involves direct interference with the bacterial ETC. Naphthoquinones intercept electrons from NADH dehydrogenase, resulting in complete respiratory arrest and the induction of cyanide-insensitive oxygen consumption. This redox cycling forces the naphthoquinone to act as an alternative electron acceptor, generating a lethal ROS surge independent of standard superoxide toxicity (Imlay and Fridovich, 1992; Medina et al., 2006). Structure-activity relationship studies confirm that hydroxyl substitutions at the C-2 or C-5 positions are critical for maximizing this redox cycling capability against multidrug-resistant pathogens (Mone et al., 2023). In biofilm microenvironments, shikonin disrupts bacterial amino acid metabolism, specifically reducing intracellular thiol concentrations to inhibit structural biofilm formation (Liu et al., 2026). The bactericidal kinetics of shikonin are amplified when coordinated with copper nanoparticles. Shikonin-copper nano-complexes achieve a 66% reduction in bacterial viability within 2 h and reduce minimum inhibitory concentrations by 50% compared to free shikonin, utilizing Cu(II)-mediated ROS generation to eradicate established biofilms (Jiang et al., 2025).

### 5.3 The metabolic awakening vulnerability

**Figure 3:**
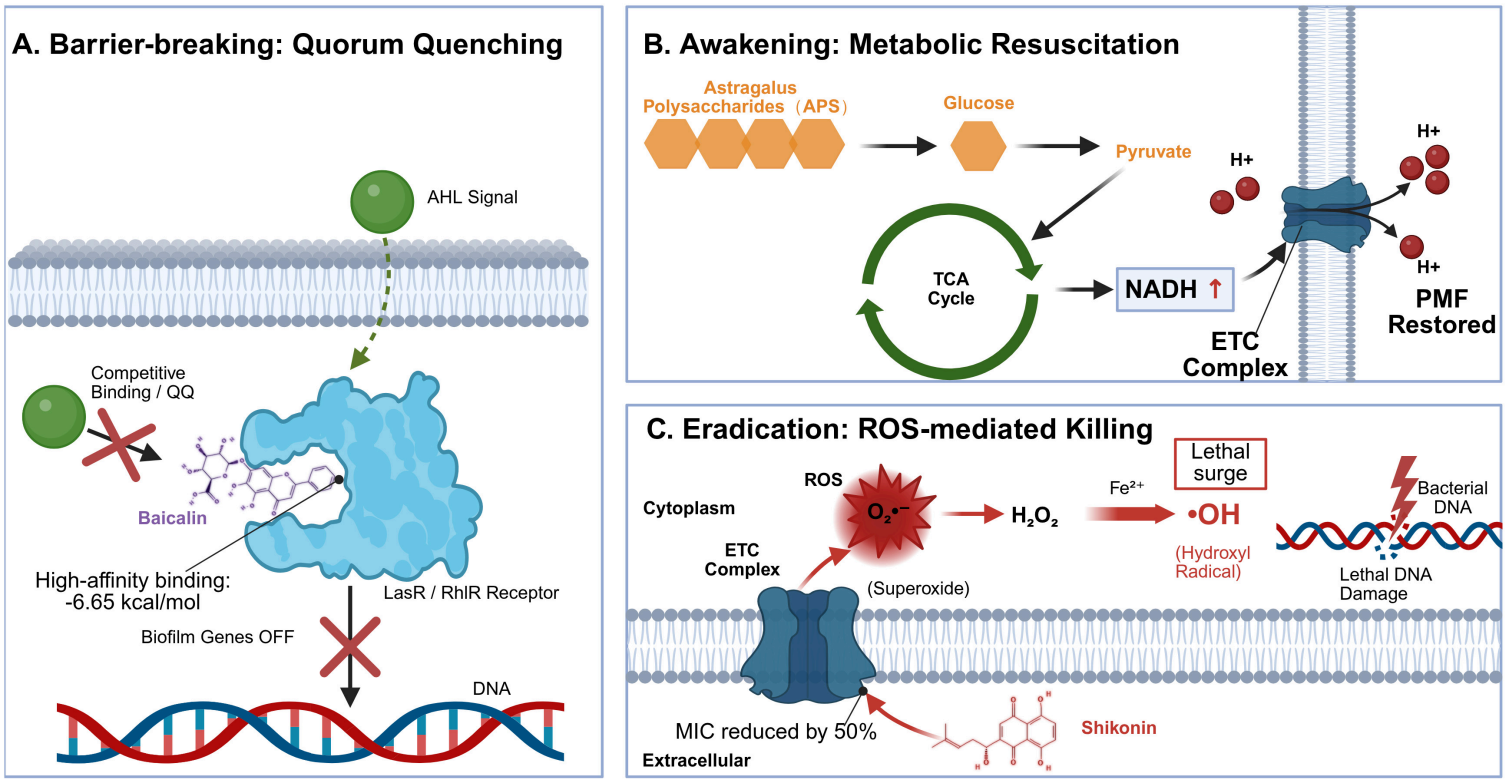
Molecular mechanisms of representative TCM-derived natural products targeting distinct persister survival pathways (created with BioRender.com). **(A)** Phase I: barrier disruption (quorum quenching). Baicalin competitively binds cytoplasmic QS receptors (e.g., LasR/RhlR), blocking native autoinducer signals (e.g., AHLs) to silence biofilm-associated virulence regulons. **(B)** Phase II: metabolic resuscitation. Within the infectious microenvironment, Astragalus polysaccharides (APS) undergo localized enzymatic hydrolysis, releasing glucose as an exogenous metabolic primer. Intracellular glucose fuels the tricarboxylic acid (TCA) cycle via pyruvate. The resulting NADH drives electron transport chain (ETC) activity, successfully regenerating the proton motive force (PMF) essential for antibiotic uptake. **(C)** Phase III: terminal eradication (ROS-Mediated Killing). Shikonin acts as a potent pro-oxidant, intercepting ETC electrons to generate superoxide anions (${\text{O}}_{2}^{-}$). These are catalyzed into highly cytotoxic hydroxyl radicals (^•^OH) via the Fenton reaction, inflicting lethal DNA damage and respiratory arrest.

Metabolically resuscitated persister cells are uniquely vulnerable to berberine and shikonin because the resuscitation process reinstates the physiological targets required for naphthoquinone and alkaloid lethality. Metabolite-driven awakening reactivates the TCA cycle, generating NADH. Elevated NADH levels feed the ETC, providing the exact substrate necessary for naphthoquinone-mediated electron interception and ROS generation (Arce-Rodríguez et al., 2022; Song et al., 2026). Active metabolism directly dictates ROS lethality; bactericidal efficacy requires elevated oxygen consumption (Dwyer et al., 2014; Grant et al., 2012). Furthermore, the resumption of macromolecular synthesis reactivates the targets for berberine's DNA gyrase and FtsZ inhibition (Roy et al., 2021; Wainwright et al., 2021). The sequential application of metabolic resuscitation followed by terminal ROS-generating agents exploits this specific physiological window, ensuring population collapse. The detailed molecular pathways driving each stage of this strategy—encompassing competitive QS receptor binding, PMF regeneration via the TCA cycle, and ETC-targeted ROS induction—are comprehensively summarized in Figure 3.

## 6 Translating concept to clinic: smart sequential delivery systems

Translating the proposed “disrupt–awaken–kill” strategy into clinical practice requires overcoming the inherent pharmacokinetic limitations of natural products, notably their low aqueous solubility, rapid systemic clearance, and poor penetration into established biofilms (Saha et al., 2026; Yang et al., 2025). To operationalize this multi-phase conceptual framework, advanced microenvironment-responsive delivery systems are required. These engineered platforms utilize the physicochemical gradients of the biofilm microenvironment—specifically acidic pH, elevated ATP concentrations, local hypoxia, and secreted bacterial enzymes—to function as precise spatiotemporal triggers for chronological drug release (Chen et al., 2023; Sousa et al., 2023; Wang Y. et al., 2023).

### 6.1 Programmed multi-layer architectures for chronological release

The sequential execution of EPS disruption (Phase I), metabolic resuscitation (Phase II), and terminal killing (Phase III) can be achieved through multi-layered core-shell particulate architectures. Proof-of-concept studies demonstrate that dual-responsive polymeric containers can orchestrate successive release profiles. For example, systems utilizing an amorphous calcium carbonate/poly(acrylic acid) (ACC/PAA) shell over an ATP-responsive zeolitic imidazolate framework-90 (ZIF-90) core effectively segregate therapeutic payloads. Similarly, step-by-step responsive systems employing pH-sensitive *cis*-aconityl-d-tyrosine (CA-Tyr) shells and enzyme-degradable polymeric cores demonstrate chronological release mechanisms *in vivo* (Fan et al., 2021).

Applying these engineering principles to the proposed TCM roadmap, a hypothetical multi-layered delivery vehicle can be conceptualized. The outermost layer, engineered for pH responsiveness and functionalized with matrix-degrading enzymes (e.g., DNase I), targets the acidic biofilm surface (Baelo et al., 2015; Deiss-Yehiely et al., 2023). Upon exposure to the pH 5.0–6.5 microenvironment, this layer undergoes charge reversal and degradation, initiating active EPS disruption and releasing Phase I agents (EGCG or baicalin) to quench QS communications. The intermediate layer, designed with ester or hyaluronic acid linkages, responds to locally concentrated bacterial enzymes (lipases or hyaluronidases) (Mohammed et al., 2023; Xiao et al., 2024). This enzymatic degradation releases Phase II agents—specifically exogenous metabolites (alanine/fumarate) and immunomodulatory APS—deep within the dispersed matrix, triggering bacterial PMF regeneration and macrophage reprogramming. Finally, the hypoxia- or ATP-responsive core accesses the deepest, previously dormant regions of the biofilm (Su et al., 2022). The elevated ATP concentrations trigger the complete dissolution of the core, releasing high local concentrations of Phase III terminal agents (berberine or shikonin) directly into the metabolically awakened bacterial population. A comprehensive profile of these representative TCM-derived agents, their precise molecular targets, and the corresponding smart delivery integration strategies across all three therapeutic phases is summarized in Table 1.

**Table 1:** Comprehensive mechanistic profiling of the TCM-derived sequential eradication strategy and smart nanocarrier integration.

Therapeutic phase	Representative TCM-derived monomers/ agents	Molecular and genetic targets	Primary pharmacological/ mechanistic action	Terminal phenotypic consequences	Smart nanocarrier integration (triggers and architecture)
Phase I: matrix disruption and quorum quenching	Epigallocatechin gallate (EGCG)	• Curli biogenesis: csgD mRNA (via RybB sRNA); RpoS (via ClpXP degradation) • Receptors: CviR, LasR, RhlR	Remodels functional amyloid (Fap) fibers into off-pathway oligomers; competitively obstructs autoinducer-receptor binding at sub-inhibitory concentrations (25–150 μg/mL).	Decreased mechanical stiffness of EPS scaffold; downregulation of pyocyanin and elastase secretion; exposure of embedded bacteria.	Trigger: acidic pH (5.0–6.5) at biofilm surface.Carrier: pH-responsive shell (e.g., ACC/PAA) functionalized with surface matrix-degrading enzymes (e.g., DNase I).
	Baicalin	• Efflux Pumps: MexAB-OprM, MsrA • QS Systems: Agr (agrA, RNAIII); AI-2 Transporter LsrB (Binding energy: −6.65 kcal/mol) • EPS Genes: icaADBC operon	Disrupts energy-dependent autoinducer export (3-oxo-C12-HSL); blocks AI-2 interspecies signal perception; halts poly-*N*-acetylglucosamine (PNAG) synthesis.	Loss of 3D biofilm architecture; prevention of early-stage microcolony aggregation; enhanced penetrability for subsequent agents.	Same as above (outer shell release layer).
Phase II: microenvironment remodeling and metabolic resuscitation	Astragalus polysaccharides (APS)	• Host macrophages: histone deacetylase 3 (HDAC3); TLR4/MyD88/TRAF-6 cascade	Modulates gut microbiota to elevate butyrate; epigenetically programs monocytes toward microbicidal M1 phenotype; stabilizes HIF-1α.	Increased localized ROS generation; enhanced LC3-associated autophagy and LL-37 production without excessive inflammatory cytokine release.	Trigger: localized bacterial enzymes (lipase, hyaluronidase, gelatinase). Carrier: enzyme-degradable intermediate layer (e.g., ester/hyaluronic acid linkages).
	Exogenous metabolites (e.g., L-alanine, fumarate)	• Bacterial respiration: TCA cycle enzymes (alanine dehydrogenase, fumarase); complex I (NADH dehydrogenase)	Drives transmembrane proton pumping via NADH-mediated electron transfer to the ETC; selectively bypasses glycolysis (fumarate via RpoN).	Rapid regeneration of the proton motive force (PMF; ΔΨ and ΔpH); active intracellular uptake of bactericidal agents restored independent of cellular division.	Same as above (intermediate release layer).
Phase III: terminal bactericidal eradication	Berberine	• Cell division: FtsZ GTPase (hydrophobic pocket binding, KD ≈ 0.023 μM) • Metabolism: carbohydrate metabolism pathways; DNA gyrase	Severely perturbs oxidative phosphorylation; destabilizes FtsZ protofilaments and disrupts *Z*-ring midcell localization; depletes intracellular γ-tocopherol.	Lethal accumulation of intracellular ROS; rapid ATP depletion; irreversible membrane depolarization via quaternary ammonium structure.	Trigger: elevated ATP concentrations and hypoxia (deep core). Carrier: ATP/hypoxia-responsive core (e.g., ZIF-90) achieving deep penetration prior to catastrophic core dissolution.
	Shikonin	• Bioenergetics: F_0_F_1_-ATP synthase (residues: αR283D, αE284R, βV265Q, γT273A); NADH dehydrogenase	Intercepts electrons from the ETC causing complete respiratory arrest; acts as an alternative electron acceptor to drive profound redox cycling.	Cyanide-insensitive oxygen consumption; massive ROS surge (hydroxyl radicals); synergistic eradication when coordinated with Cu(II) nanoparticles.	Same as above (deep core payload).

### 6.2 Overcoming bioavailability and delivery barriers

These smart delivery systems directly resolve the bioavailability barriers that currently limit TCM monomer application. Advanced encapsulation of berberine, for instance, significantly enhances its pharmacokinetic profile. Berberine-loaded gelatin formulations and micellar systems demonstrate biphasic sustained release over extended periods, elevating local minimum inhibitory concentrations without systemic toxicity (Cheng et al., 2023; Sharaf et al., 2026; Virzì et al., 2024). Furthermore, combinatorial liposomes co-encapsulating multiple agents exhibit synergistic fractional inhibitory concentration indices (FICI), reducing the required dose of free compounds by up to 96%(Bhatia et al., 2021). Furthermore, niosome encapsulation of curcumin resolves its intrinsic solubility limitations and actively downregulates the icaA gene involved in biofilm development (Khaleghian et al., 2023). Advanced GSH/pH cascade-responsive nanoparticles further exploit the glutathione-rich biofilm microenvironment; disulfide bond cleavage triggers rapid size reduction for deep penetration, enabling synergistic photo-chemo eradication of multidrug-resistant strains (Kang et al., 2024).

Advanced cascade systems further augment penetration capabilities. Nanocarriers utilizing glucose oxidase-mimic activity and l-arginine generate nitric oxide (NO), providing self-propelled penetration into deep biofilm layers (Zheng et al., 2022). Alternatively, near-infrared (NIR) triggered systems generate ROS and NO cascade reactions to induce simultaneous matrix dispersal and localized bactericidal activity (Zou et al., 2023). By integrating the distinct pharmacological properties of TCM-derived agents with sophisticated, stimuli-responsive formulation technologies, the theoretical multi-phase sequential therapy can be translated into a clinically feasible, single-formulation delivery system capable of completely eradicating biofilm-associated persister infections.

## 7 Conclusion and translational perspectives

The eradication of biofilm-associated persister cells requires interventions that address both structural barriers and metabolic dormancy. Conventional monotherapies targeting active cellular processes exhibit profound limitations against these protected subpopulations. This review outlines a multi-phase sequential framework: extracellular matrix disruption, metabolic resuscitation, and terminal bactericidal killing. Plant-derived secondary metabolites provide specific pharmacological mechanisms for executing this framework. EGCG and baicalin degrade the EPS matrix and inhibit quorum sensing. APS and exogenous metabolites (such as l-alanine and fumarate) restore host immune function and regenerate the bacterial proton motive force. Subsequently, berberine and shikonin exploit this reactivated metabolic state, inducing lethal ROS accumulation and irreversible respiratory arrest. Microenvironment-responsive delivery architectures translate this multi-agent strategy into a clinically viable delivery system. Stimuli-responsive platforms utilize biofilm-specific physicochemical gradients—acidic pH, elevated ATP, and bacterial enzymes—to achieve precise spatiotemporal control over chronological drug release.

## 8 Manufacturing scale-up and reproducibility

Despite robust preclinical evidence, the clinical translation of multi-component botanical formulations faces formidable manufacturing challenges. Batch-to-batch variability remains a primary limitation for multi-layered carriers (Dormont et al., 2019). Industrial scale-up processes frequently alter nanoparticle size, surface charge, and protein adsorption characteristics compared to laboratory-scale synthesis, as demonstrated in squalene-based and poly(lactic-co-glycolic acid) (PLGA) systems (Dormont et al., 2019; Operti et al., 2021). Furthermore, the layer-by-layer electrostatic assembly required for sequential release formulations necessitates multiple purification steps that significantly reduce yield and efficiency (Correa et al., 2016). Implementing continuous manufacturing processes, such as inline sonication combined with tangential flow filtration, alongside microfluidic-mediated assembly, is required to improve batch consistency and analytical scalability (Operti et al., 2022; Pires et al., 2025).

## 9 Regulatory complexity

There is currently no globally harmonized regulatory framework for multicomposite, multifunctional therapeutic systems (Mangla et al., 2025; Rodríguez-Gómez et al., 2024). The standard generic “sameness” paradigm is inappropriate for complex carriers due to their inherent structural heterogeneity; regulatory agencies increasingly require a stepwise similarity approach evaluating critical quality attributes (CQAs) and non-clinical biodistribution profiles (Hussaarts et al., 2017; Mühlebach, 2018). Furthermore, botanical drug products present unique characterization challenges. The FDA has approved only two botanical new drug applications (NDAs) to date, reflecting the intense scientific difficulty of ensuring quality consistency and demonstrating combination plausibility for multi-herbal formulations (Qu et al., 2022; Wu et al., 2020).

## 10 Clinical trial attrition

Clinical translation exhibits high attrition rates. Comprehensive analysis of advanced drug delivery trials reveals a phase 3 success rate of approximately 14%, with late-stage failures predominantly attributed to a lack of clinical efficacy rather than host toxicity (He et al., 2019). Anti-biofilm targeted formulations specifically lack FDA-approved formulations, with the vast majority of current studies restricted strictly to *in vitro* validation (Al-Wrafy et al., 2022). Additionally, translating *in vitro* synergistic interactions (e.g., fractional inhibitory concentration indices) into *in vivo* efficacy remains highly unpredictable due to the lack of standardized, clinically relevant synergy testing methodologies (Gómara and Ramón-García, 2019; Karakonstantis et al., 2022).

## 11 Future directions

Advancing these therapeutic strategies requires the strict adoption of Quality by Design (QbD) principles to establish robust manufacturing design spaces and real-time process monitoring (Csóka et al., 2021). Integrating artificial intelligence tools, such as generative adversarial networks, can optimize clinical trial design by systematically bridging preclinical and clinical datasets (Khan et al., 2025). Collaborative efforts among researchers, pharmaceutical stakeholders, and regulatory authorities are essential to establish standardized characterization techniques and accelerate the clinical deployment of sequential anti-biofilm therapies.

## References

[B1] Alboslemy T. Yu B. Rogers T. Kim M. (2019). *Staphylococcus aureus* biofilm-conditioned medium impairs macrophage-mediated antibiofilm immune response by upregulating klf2 expression. Infect. Immun. 87, e618–e643. doi: 10.1128/IAI.00643-18 PMC6434135 30692179

[B2] Allison K. R. Brynildsen M. P. Collins J. J. (2011). Metabolite-enabled eradication of bacterial persisters by aminoglycosides. Nature 473, 216–220. doi: 10.1038/nature10069 21562562 PMC3145328

[B3] Al-Wrafy F. A. Al-Gheethi A. A. Ponnusamy S. K. Noman E. A. Fattah S. A. (2022). Nanoparticles approach to eradicate bacterial biofilm-related infections: a critical review. Chemosphere 288(Pt 2):132603. doi: 10.1016/j.chemosphere.2021.132603 34678351

[B4] An E. Zhang W. Kwak M. Lee P. C. Jin J. (2022). Polysaccharides from astragalus membranaceus elicit t cell immunity by activation of human peripheral blood dendritic cells. Int. J. Biol. Macromol. 223(Pt A), 370–377. doi: 10.1016/j.ijbiomac.2022.11.048 36368354

[B5] Arce-Rodríguez A. Pankratz D. Preusse M. Nikel P. I. Häussler S. (2022). Dual effect: high nadh levels contribute to efflux-mediated antibiotic resistance but drive lethality mediated by reactive oxygen species. Mbio 13, e2421–e2434. doi: 10.1128/mbio.02434-21 PMC8764520 35038918

[B6] Arita-Morioka K. Yamanaka K. Mizunoe Y. Tanaka Y. Ogura T. Sugimoto S. (2018). Inhibitory effects of myricetin derivatives on curli-dependent biofilm formation in *Escherichia coli*. Sci. Rep. 8:8452. doi: 10.1038/s41598-018-26748-z 29855532 PMC5981455

[B7] Baelo A. Levato R. Julián E. Crespo A. Astola J. Gavaldà J. . (2015). Disassembling bacterial extracellular matrix with dnase-coated nanoparticles to enhance antibiotic delivery in biofilm infections. J. Control. Release. 209, 150–158. doi: 10.1016/j.jconrel.2015.04.028 25913364

[B8] Bakkeren E. Diard M. Hardt W. (2020). Evolutionary causes and consequences of bacterial antibiotic persistence. Nat. Rev. Microbiol. 18, 479–490. doi: 10.1038/s41579-020-0378-z 32461608

[B9] Bao X. Bové M. Coenye T. (2022). Organic acids and their salts potentiate the activity of selected antibiotics against pseudomonas aeruginosa biofilms grown in a synthetic cystic fibrosis sputum medium. Antimicrob. Agents. Chemother. 66, e1821–e1875. doi: 10.1128/AAC.01875-21 PMC8765270 34807756

[B10] Batchelder J. I. Taylor A. J. Mok W. W. K. (2024). Metabolites augment oxidative stress to sensitize antibiotic-tolerant *Staphylococcus aureus* to fluoroquinolones. Mbio 15:e271424. doi: 10.1128/mbio.02714-24 PMC11633220 39475229

[B11] Beam J. E. Wagner N. J. Shook J. C. Bahnson E. S. M. Fowler V. G. Rowe S. E. . (2021). Macrophage-produced peroxynitrite induces antibiotic tolerance and supersedes intrinsic mechanisms of persister formation. Infect. Immun. 89, e221–e286. doi: 10.1128/IAI.00286-21 PMC8445188 34097475

[B12] Bhatia E. Sharma S. Jadhav K. Banerjee R. (2021). Combinatorial liposomes of berberine and curcumin inhibit biofilm formation and intracellular methicillin resistant *staphylococcus aureus* infections and associated inflammation. J. Mat. Chem. B. 9, 864–875. doi: 10.1039/D0TB02036B 33392614

[B13] Boberek J. M. Stach J. Good L. (2010). Genetic evidence for inhibition of bacterial division protein ftsz by berberine. PLoS One 5:e13745. doi: 10.1371/journal.pone.0013745 21060782 PMC2966414

[B14] Böhning J. Tarafder A. K. Bharat T. A. M. (2024). The role of filamentous matrix molecules in shaping the architecture and emergent properties of bacterial biofilms. Biochem. J. 481, 245–263. doi: 10.1042/BCJ20210301 38358118 PMC10903470

[B15] Bryson D. Hettle A. G. Boraston A. B. Hobbs J. K. (2020). Clinical mutations that partially activate the stringent response confer multidrug tolerance instaphylococcus aureus. Antimicrob. Agents. Chemother. 64, e2103–e2119. doi: 10.1128/AAC.02103-19 PMC7038295 31871080

[B16] Cai Y. Si Z. Jiang Y. Ye M. Wang F. Yang X. . (2023). Structure-activity relationship of low molecular weight astragalus membranaceus polysaccharides produced by bacteroides. Carbohydr. Polym. 316:121036. doi: 10.1016/j.carbpol.2023.121036 37321731

[B17] Chen Y. Gao Y. Huang Y. Jin Q. Ji J. (2023). Inhibiting quorum sensing by active targeted ph-sensitive nanoparticles for enhanced antibiotic therapy of biofilm-associated bacterial infections. ACS Nano 17, 10019–10032. doi: 10.1021/acsnano.2c12151 37234036

[B18] Chen Y. Liu T. Wang K. Hou C. Cai S. Huang Y. . (2016). Baicalein inhibits *Staphylococcus aureus* biofilm formation and the quorum sensing system in vitro. PLoS One 11:e153468. doi: 10.1371/journal.pone.0153468 PMC4851419 27128436

[B19] Chen Z. Gao Y. Lv B. Sun F. Yao W. Wang Y. . (2019). Hypoionic shock facilitates aminoglycoside killing of both nutrient shift- and starvation-induced bacterial persister cells by rapidly enhancing aminoglycoside uptake. Front. Microbiol. 10:2028. doi: 10.3389/fmicb.2019.02028 31551965 PMC6743016

[B20] Cheng G. Jian S. Li W. Yan L. Chen T. Cheng T. . (2024). Epigallocatechin gallate protects mice from salmonella enterica ser. *Typhimurium* infection by modulating bacterial virulence through quorum sensing inhibition. Front. Cell. Infect. Microbiol. 14:1432111. doi: 10.3389/fcimb.2024.1432111 39479281 PMC11521958

[B21] Cheng X. Geng J. Wang L. Ma X. Su Y. Arif M. . (2023). Berberine-loaded mannosylerythritol lipid-b nanomicelles as drug delivery carriers for the treatment of helicobacter pylori biofilms in vivo. Eur. J. Pharm. Biopharm. 193, 105–118. doi: 10.1016/j.ejpb.2023.10.021 39492446

[B22] Correa S. Choi K. Y. Dreaden E. C. Renggli K. Shi A. Gu L. . (2016). Highly scalable, closed–loop synthesis of drug–loaded, layer–by–layer nanoparticles. Adv. Funct. Mater. 26, 991–1003. doi: 10.1002/adfm.201504385 27134622 PMC4847955

[B23] Crabbé A. Ostyn L. Staelens S. Rigauts C. Risseeuw M. Dhaenens M. . (2019). Host metabolites stimulate the bacterial proton motive force to enhance the activity of aminoglycoside antibiotics. PLoS Pathog. 15:e1007697. doi: 10.1371/journal.ppat.1007697 31034512 PMC6508747

[B24] Csóka I. Ismail R. Jójárt-Laczkovich O. Pallagi E. (2021). Regulatory considerations, challenges and risk-based approach in nanomedicine development. Curr. Med. Chem. 28, 7461–7476. doi: 10.2174/0929867328666210406115529 33823761

[B25] Deiss-Yehiely E. Cárcamo-Oyarce G. Berger A. G. Ribbeck K. Hammond P. T. (2023). Ph-responsive, charge-reversing layer-by-layer nanoparticle surfaces enhance biofilm penetration and eradication. Acs Biomater. Sci. Eng. 9, 4794–4804. doi: 10.1021/acsbiomaterials.3c00481 37390118 PMC11117027

[B26] Domadia P. N. Bhunia A. Sivaraman J. Swarup S. Dasgupta D. (2008). Berberine targets assembly of *Escherichia coli* cell division protein ftsz. Biochemistry 47, 3225–3234. doi: 10.1021/bi7018546 18275156

[B27] Domouchtsidou A. Ioannou P. Lianou A. Tsante K. A. Tsakri D. Bonova E. . (2026). Biofilms in clinical infection: pathophysiology, diagnosis, and the evolving therapeutic landscape. J. Clin. Microbiol. 64:e104225. doi: 10.1128/jcm.01042-25 PMC12977598 41406024

[B28] Donnert M. Elsheikh S. Arce-Rodriguez A. Pawar V. Braubach P. Jonigk D. . (2020). Targeting bioenergetics is key to counteracting the drug-tolerant state of biofilm-grown bacteria. PLoS Pathog. 16:e1009126. doi: 10.1371/journal.ppat.1009126 33351859 PMC7787680

[B29] Dormont F. Rouquette M. Mahatsekake C. Gobeaux F. Peramo A. Brusini R. . (2019). Translation of nanomedicines from lab to industrial scale synthesis: the case of squalene-adenosine nanoparticles. J. Control. Release. 307, 302–314. doi: 10.1016/j.jconrel.2019.06.040 31260754

[B30] Du G. Le Y. Sun X. Yang X. He Q. (2020). Proteomic investigation into the action mechanism of berberine against streptococcus pyogenes. J. Proteomics 215:103666. doi: 10.1016/j.jprot.2020.103666 31981716

[B31] Dwyer D. J. Belenky P. A. Yang J. H. MacDonald I. C. Martell J. D. Takahashi N. . (2014). Antibiotics induce redox-related physiological alterations as part of their lethality. Proc. Natl. Acad. Sci. U. S. A. 111, E2100–E2109. doi: 10.1073/pnas.1401876111 24803433 PMC4034191

[B32] Fakhar M. Ahmed M. Nasim Sabri A. (2024). Computational and experimental strategies for combating mbl p. Aeruginosa (mblpa) biofilms using phytochemicals: targeting the quorum sensing network. Saudi J. Biol. Sci. 31:104001. doi: 10.1016/j.sjbs.2024.104001 38646565 PMC11031748

[B33] Fan Q. Wang C. Guo R. Jiang X. Li W. Chen X. . (2021). Step-by-step dual stimuli-responsive nanoparticles for efficient bacterial biofilm eradication. Biomater. Sci. 9, 6889–6902. doi: 10.1039/D1BM01038G 34519743

[B34] Fisher R. A. Gollan B. Helaine S. (2017). Persistent bacterial infections and persister cells. Nat. Rev. Microbiol. 15, 453–464. doi: 10.1038/nrmicro.2017.42 28529326

[B35] Gollan B. Grabe G. Michaux C. Helaine S. (2019). Bacterial persisters and infection: past, present, and progressing. Annu. Rev. Microbiol. 73, 359–385. doi: 10.1146/annurev-micro-020518-115650 31500532

[B36] Gómara M. Ramón-García S. (2019). The fici paradigm: correcting flaws in antimicrobial in vitro synergy screens at their inception. Biochem. Pharmacol. 163, 299–307. doi: 10.1016/j.bcp.2019.03.001 30836058

[B37] Gon Alves A. S. C. Leit O M. M. Sim Es M. Borges A. (2023). The action of phytochemicals in biofilm control. Nat. Prod. Rep. 40, 595–627. doi: 10.1039/D2NP00053A 36537821

[B38] Grant S. S. Kaufmann B. B. Chand N. S. Haseley N. Hung D. T. (2012). Eradication of bacterial persisters with antibiotic-generated hydroxyl radicals. Proc. Nat. Acad. Sci. 109, 12147–12152. doi: 10.1073/pnas.1203735109 22778419 PMC3409745

[B39] Hall C. W. Farkas E. Zhang L. Mah T. (2019). Potentiation of aminoglycoside lethality by c4 -dicarboxylates requires rpon in antibiotic-tolerant pseudomonas aeruginosa. Antimicrob. Agents. Chemother. 63, e1313–e1319. doi: 10.1128/AAC.01313-19 PMC6761562 31383655

[B40] Hall C. W. Mah T. (2017). Molecular mechanisms of biofilm-based antibiotic resistance and tolerance in pathogenic bacteria. Fems Microbiol. Rev. 41, 276–301. doi: 10.1093/femsre/fux010 28369412

[B41] Hanke M. L. Heim C. E. Angle A. Sanderson S. D. Kielian T. (2013). Targeting macrophage activation for the prevention and treatment of *Staphylococcus aureus* biofilm infections. J. Immunol. 190, 2159–2168. doi: 10.4049/jimmunol.1202348 23365077 PMC3578052

[B42] Harms A. Maisonneuve E. Gerdes K. (2016). Mechanisms of bacterial persistence during stress and antibiotic exposure. Science 354:aaf4268. doi: 10.1126/science.aaf4268 27980159

[B43] He H. Liu L. Morin E. E. Liu M. Schwendeman A. (2019). Survey of clinical translation of cancer nanomedicines—lessons learned from successes and failures. Acc. Chem. Res. 52, 2445–2461. doi: 10.1021/acs.accounts.9b00228 31424909

[B44] Høiby N. Bjarnsholt T. Moser C. Bassi G. L. Coenye T. Donelli G. . (2015). Escmid* guideline for the diagnosis and treatment of biofilm infections 2014. Clin. Microbiol. Infect. 21, S1–S25. doi: 10.1016/j.cmi.2014.10.024 25596784

[B45] Huo Z. Li J. Li X. Xiao H. Lin Y. Ma Y. . (2024). Functional fractions of astragalus polysaccharides as a potential prebiotic to alleviate ulcerative colitis. Int. J. Biol. Macromol. 271(Pt 1):132580. doi: 10.1016/j.ijbiomac.2024.132580 38788871

[B46] Hussaarts L. Mühlebach S. Shah V. P. McNeil S. Borchard G. Flühmann B. . (2017). Equivalence of complex drug products: advances in and challenges for current regulatory frameworks. Ann. N. Y. Acad. Sci. 1407, 39–49. doi: 10.1111/nyas.13347 28445611

[B47] Imlay J. Fridovich I. (1992). Exogenous quinones directly inhibit the respiratory nadh dehydrogenase in *Escherichia coli*. Arch. Biochem. Biophys. 296, 337–346. doi: 10.1016/0003-9861(92)90581-G 1318694

[B48] Jiang Y. Tang X. Wang A. Yang H. Jiang X. Zhang Y. . (2025). Shikonin-copper coordination nanoparticles for enhanced antibacterial and antibiofilm activity against *Staphylococcus aureus*. Sci. Rep. 15:39617. doi: 10.1038/s41598-025-23269-4 41224798 PMC12612078

[B49] Kang X. Yang X. Bu F. Feng W. Liu F. Xie W. . (2024). Gsh/ph cascade-responsive nanoparticles eliminate methicillin-resistant *Staphylococcus aureus* biofilm via synergistic photo-chemo therapy. ACS Appl. Mater. Interfaces 16, 3202–3214. doi: 10.1021/acsami.3c17198 38207171

[B50] Karakonstantis S. Ioannou P. Kofteridis D. D. (2022). In search for a synergistic combination against pandrug-resistant a. Baumannii; Methodological considerations. Infection 50, 569–581. doi: 10.1007/s15010-021-01748-w 34982411

[B51] Khaleghian M. Sahrayi H. Hafezi Y. Mirshafeeyan M. Moghaddam Z. S. Farasati Far B. . (2023). In silico design and mechanistic study of niosome-encapsulated curcumin against multidrug-resistant *Staphylococcus aureus* biofilms. Front. Microbiol. 14:1277533. doi: 10.3389/fmicb.2023.1277533 38098658 PMC10720333

[B52] Khan S. Gull A. Akhtar M. Gull B. Najmi A. K. Parveen R. . (2025). Explicit analysis of in vivo, meterological and statistical hurdles in successful clinical translation of targeted nanomedicines and plausible remedial strategies. Expert Opin. Drug Deliv. 22, 1769–1791. doi: 10.1080/17425247.2025.2556979 40913551

[B53] Kim B. ParK J. Choi H. Kwak J. Kim W. (2019). Differential effects of alkyl gallates on quorum sensing in pseudomonas aeruginosa. Sci. Rep. 9:7741. doi: 10.1038/s41598-019-44236-w 31123307 PMC6533263

[B54] Kim S. B. Lyou E. S. Kim M. S. Lee T. K. (2023). Bacterial resuscitation from starvation-induced dormancy results in phenotypic diversity coupled with translational activity depending on carbon substrate availability. Microb. Ecol. 86, 325–336. doi: 10.1007/s00248-022-02068-8 35788867

[B55] Kitzenberg D. A. Lee J. S. Mills K. B. Kim J. Liu L. Vázquez-Torres A. . (2022). Adenosine awakens metabolism to enhance growth-independent killing of tolerant and persister bacteria across multiple classes of antibiotics. Mbio 13, e422–e480. doi: 10.1128/mbio.00480-22 PMC9239199 35575513

[B56] Koo H. Allan R. N. Howlin R. P. Stoodley P. Hall-Stoodley L. (2017). Targeting microbial biofilms: current and prospective therapeutic strategies. Nat. Rev. Microbiol. 15, 740–755. doi: 10.1038/nrmicro.2017.99 28944770 PMC5685531

[B57] Lee Y. Lee D. Kim Y. B. Lee S. Cha S. Park H. . (2015). The mechanism underlying the antibacterial activity of shikonin against methicillin-resistant *Staphylococcus aureus*. Evid. Based Complement. Altern. Med. 2015, 1–9. doi: 10.1155/2015/520578 PMC4523682 26265924

[B58] Li H. Fan D. Liu X. Chen Y. Xiao J. Hou C. . (2026). Stage-specific therapeutic strategies for combating bacterial biofilm infections: recent advances and future perspectives. J. Control. Release. 393:114815. doi: 10.1016/j.jconrel.2026.114815 41831689

[B59] Li K. Li X. Li G. Cui L. Qin X. Li Z. . (2022). Relationship between the structure and immune activity of components from the active polysaccharides aps-ii of astragali radix by enzymolysis of endo α-1,4-glucanase. Front. Pharmacol. 13:839635. doi: 10.3389/fphar.2022.839635 35281923 PMC8913491

[B60] Li L. Chen X. Lv M. Cheng Z. Liu F. Wang Y. . (2022). Effect of platycodon grandiflorus polysaccharide on m1 polarization induced by autophagy degradation of socs1/2 proteins in 3d4/21 cells. Front. Immunol. 13:934084. doi: 10.3389/fimmu.2022.934084 35844489 PMC9279577

[B61] Li S. Pang J. Liu Y. He X. Wang Y. Wang S. . (2025). Baicalin inhibits conjugative transfer of multidrug-resistant plasmid rp4 by regulating autoinducer-2 transporter protein lsrb. Ecotoxicol. Environ. Saf. 298:118334. doi: 10.1016/j.ecoenv.2025.118334 40378727

[B62] Liu Q. Tang Y. Jiang S. Yu X. Zhu H. Xie X. . (2024). Mechanisms of action of berberine hydrochloride in planktonic cells and biofilms of pseudomonas aeruginosa. Microb. Pathog. 193:106774. doi: 10.1016/j.micpath.2024.106774 38969184

[B63] Liu Q. Yao Y. Zhang S. Sheng Z. (2011). Astragalus polysaccharides regulate t cell-mediated immunity via cd11chighcd45rblow dcs in vitro. J. Ethnopharmacol. 136, 457–464. doi: 10.1016/j.jep.2010.06.041 20620204

[B64] Liu S. Zhang H. Zheng X. Mou X. Wu Z. Zou L. . (2026). Shikonin inhibits mrsa biofilm formation to alleviate periprosthetic joint infection. Front. Pharmacol. 17:1739888. doi: 10.3389/fphar.2026.1739888 41868125 PMC13002819

[B65] Liu T. Sun Z. Yang Z. Qiao X. (2023). Microbiota-derived short-chain fatty acids and modulation of host-derived peptides formation: focused on host defense peptides. Biomed. Pharmacother. 162:114586. doi: 10.1016/j.biopha.2023.114586 36989711

[B66] Liu Y. Yang K. Jia Y. Shi J. Tong Z. Wang Z. (2021). Thymine sensitizes gram-negative pathogens to antibiotic killing. Front. Microbiol. 12:622798. doi: 10.3389/fmicb.2021.622798 33584625 PMC7875874

[B67] Lu K. Yang X. Eldridge M. J. G. Sun R. Giorgio R. T. Morris B. I. . (2025). A host-directed adjuvant sensitizes intracellular bacterial persisters to antibiotics. Nat. Microbiol. 10, 3013–3025. doi: 10.1038/s41564-025-02124-2 41073665 PMC12578635

[B68] Luo J. Dong B. Wang K. Cai S. Liu T. Cheng X. . (2017). Baicalin inhibits biofilm formation, attenuates the quorum sensing-controlled virulence and enhances pseudomonas aeruginosa clearance in a mouse peritoneal implant infection model. PLoS One 12:e176883. doi: 10.1371/journal.pone.0176883 PMC5409170 28453568

[B69] Luo T. Lu Y. Yan S. Xiao X. Rong X. Guo J. (2020). Network pharmacology in research of chinese medicine formula: methodology, application and prospective. Chin. J. Integr. Med. 26, 72–80. doi: 10.1007/s11655-019-3064-0 30941682

[B70] Mangla B. Kumar P. Javed S. Pathan T. Ahsan W. Aggarwal G. (2025). Regulating nanomedicines: challenges, opportunities, and the path forward. Nanomedicine 20, 1911–1927. doi: 10.1080/17435889.2025.2533107 40657903 PMC12320824

[B71] Marques C. N. H. Morozov A. Planzos P. Zelaya H. M. (2014). The fatty acid signaling moleculecis−2-decenoic acid increases metabolic activity and reverts persister cells to an antimicrobial-susceptible state. Appl. Environ. Microbiol. 80, 6976–6991. doi: 10.1128/AEM.01576-14 25192989 PMC4249009

[B72] Medina L. F. C. Hertz P. F. Stefani V. Henriques J. A. P. Zanotto-Filho A. Brandelli A. (2006). Aminonaphthoquinone induces oxidative stress instaphylococcus aureus. Biochem. Cell. Biol. 84, 720–727. doi: 10.1139/o06-087 17167535

[B73] Mohammed M. Ibrahim U. H. Aljoundi A. Omolo C. A. Devnarain N. Gafar M. A. . (2023). Enzyme-responsive biomimetic solid lipid nanoparticles for antibiotic delivery against hyaluronidase-secreting bacteria. Int. J. Pharm. 640:122967. doi: 10.1016/j.ijpharm.2023.122967 37084831

[B74] Mone N. S. Syed S. Ravichandiran P. Satpute S. K. Kim A. R. Yoo D. J. (2023). How structure–function relationships of 1,4–naphthoquinones combat antimicrobial resistance in multidrug–resistant (mdr) pathogens. Chemmedchem 18:e202200471. doi: 10.1002/cmdc.202200471 36316281

[B75] Mühlebach S. (2018). Regulatory challenges of nanomedicines and their follow-on versions: a generic or similar approach? Adv. Drug Deliv. Rev. 131, 122–131. doi: 10.1016/j.addr.2018.06.024 29966685

[B76] Müsken M. Pawar V. Schwebs T. Bähre H. Felgner S. Weiss S. . (2018). Breaking the vicious cycle of antibiotic killing and regrowth of biofilm-residingpseudomonas aeruginosa. Antimicrob. Agents. Chemother. 62, e1618–e1635. doi: 10.1128/AAC.01635-18 PMC6256772 30297365

[B77] Najarzadeh Z. Mohammad-Beigi H. Nedergaard Pedersen J. Christiansen G. Sønderby T. V. Shojaosadati S. A. . (2019). Plant polyphenols inhibit functional amyloid and biofilm formation in pseudomonas strains by directing monomers to off-pathway oligomers. Biomolecules 9:659. doi: 10.3390/biom9110659 31717821 PMC6920965

[B78] Operti M. C. Bernhardt A. Grimm S. Engel A. Figdor C. G. Tagit O. (2021). Plga-based nanomedicines manufacturing: technologies overview and challenges in industrial scale-up. Int. J. Pharm. 605:120807. doi: 10.1016/j.ijpharm.2021.120807 34144133

[B79] Operti M. C. Bernhardt A. Sincari V. Jager E. Grimm S. Engel A. . (2022). Industrial scale manufacturing and downstream processing of plga-based nanomedicines suitable for fully continuous operation. Pharmaceutics 14:276. doi: 10.3390/pharmaceutics14020276 35214009 PMC8878443

[B80] Orman M. A. Brynildsen M. P. (2013). Establishment of a method to rapidly assay bacterial persister metabolism. Antimicrob. Agents. Chemother. 57, 4398–4409. doi: 10.1128/AAC.00372-13 23817376 PMC3754326

[B81] Patel H. Buchad H. Gajjar D. (2022). Pseudomonas aeruginosa persister cell formation upon antibiotic exposure in planktonic and biofilm state. Sci. Rep. 12:16151. doi: 10.1038/s41598-022-20323-3 36168027 PMC9515113

[B82] Peng B. Su Y. Li H. Han Y. Guo C. Tian Y. . (2015). Exogenous alanine and/or glucose plus kanamycin kills antibiotic-resistant bacteria. Cell Metab. 21, 249–262. doi: 10.1016/j.cmet.2015.01.008 25651179

[B83] Peng L. Yuan M. Wu Z. Song K. Zhang C. An Q. . (2019). Anti-bacterial activity of baicalin against apec through inhibition of quorum sensing and inflammatory responses. Sci. Rep. 9:4063. doi: 10.1038/s41598-019-40684-6 30858423 PMC6411720

[B84] Peyrusson F. Nguyen T. K. Najdovski T. Van Bambeke F. (2022). Host cell oxidative stress induces dormant *Staphylococcus aureus* persisters. Microbiol. Spectr. 10, e2313–e2321. doi: 10.1128/spectrum.02313-21 PMC8865412 35196815

[B85] Pinto R. M. Soares F. A. Reis S. Nunes C. Van Dijck P. (2020). Innovative strategies toward the disassembly of the eps matrix in bacterial biofilms. Front. Microbiol. 11:952. doi: 10.3389/fmicb.2020.00952 32528433 PMC7264105

[B86] Pires I. S. Gordon E. Suh H. Irvine D. J. Hammond P. T. (2025). High–throughput microfluidic–mediated assembly of layer–by–layer nanoparticles. Adv. Funct. Mater. 35:2503965. doi: 10.1002/adfm.202503965 PMC12463190 41019011

[B87] Prax M. Mechler L. Weidenmaier C. Bertram R. (2016). Glucose augments killing efficiency of daptomycin challenged *Staphylococcus aureus* persisters. PLoS One 11:e150907. doi: 10.1371/journal.pone.0150907 PMC4784881 26960193

[B88] Qu L. Li X. Xiong Y. Wang Z. Zhou Y. Zou W. . (2022). Opportunities and hurdles to european market access for multi-herbal traditional chinese medicine products: an analysis of eu regulations for combination herbal medicinal products. Pharmacol. Res. 186:106528. doi: 10.1016/j.phrs.2022.106528 36332812

[B89] Rodríguez-Gómez F. D. Monferrer D. Penon O. Rivera-Gil P. (2024). Implementing horizon scanning as a tool for the strategic development of regulatory guidelines for nanotechnology-enabled health products. Front. Med. 10:1308047. doi: 10.3389/fmed.2023.1308047 PMC10829765 38298514

[B90] Rong X. Zhu L. Shu Q. (2025). Synergistic gut microbiome-mediated degradation of astragalus membranaceus polysaccharides and codonopsis pilosula polysaccharides into butyric acid: a metatranscriptomic analysis. Microbiol. Spectr. 13:e303924. doi: 10.1128/spectrum.03039-24 PMC12210856 40422281

[B91] Roy S. Bahar A. A. Gu H. Nangia S. Sauer K. Ren D. (2021). Persister control by leveraging dormancy associated reduction of antibiotic efflux. PLoS Pathog. 17:e1010144. doi: 10.1371/journal.ppat.1010144 34890435 PMC8716142

[B92] Ruiz-Sorribas A. Poilvache H. Kamarudin N. H. N. Braem A. Van Bambeke F. (2022). Hydrolytic enzymes as potentiators of antimicrobials against an inter-kingdom biofilm model. Microbiol. Spectr. 10, e2521–e2589. doi: 10.1128/spectrum.02589-21 PMC8865531 35196793

[B93] Saha S. Roy A. Gill H. S. Rajeev M. Nag M. Pandit S. . (2026). Berberine as a therapeutic alkaloid against eskape and multiple drug-resistant bacteria: a comprehensive review. Arch. Microbiol. 208:115. doi: 10.1007/s00203-025-04663-y 41524767

[B94] Schulthess J. Pandey S. Capitani M. Rue-Albrecht K. C. Arnold I. Franchini F. . (2019). The short chain fatty acid butyrate imprints an antimicrobial program in macrophages. Immunity 50, 432–445. doi: 10.1016/j.immuni.2018.12.018 30683619 PMC6382411

[B95] Serra D. O. Mika F. Richter A. M. Hengge R. (2016). The green tea polyphenol EGCG inhibits *E. coli* biofilm formation by impairing amyloid curli fibre assembly and downregulating the biofilm regulator CsgD via the σ^E^-dependent sRNA RybB. Mol. Microbiol. 101, 136–151. doi: 10.1111/mmi.13379 26992034

[B96] Shan Y. Brown Gandt A. Rowe S. E. Deisinger J. P. Conlon B. P. Lewis K. (2017). Atp-dependent persister formation in *Escherichia coli*. Mbio 8, e2216–e2267. doi: 10.1128/mBio.02267-16 PMC5296605 28174313

[B97] Sharaf H. A. Abu Saied M. A. Ghareeb D. A. Kandil S. H. El-Fattah A. A. (2026). Enhanced controlled drug delivery of berberine-loaded gelatin nanoparticles: characterization andin vitro assessment. RSC Adv. 16, 6119–6131. doi: 10.1039/D5RA08567E 41624931 PMC12853093

[B98] Sharma S. Mohler J. Mahajan S. D. Schwartz S. A. Bruggemann L. Aalinkeel R. (2023). Microbial biofilm: a review on formation, infection, antibiotic resistance, control measures, and innovative treatment. Microorganisms 11:1614. doi: 10.3390/microorganisms11061614 37375116 PMC10305407

[B99] Sikdar B. Paul D. Banik S. K. Dastidar S. G. Gangopadhyay G. (2026). An integrated in-vitro, transcriptomic, and in-silico approach to understand the molecular mechanism of quorum-sensing inhibition by epigallocatechin-3-gallate (egcg) in chromobacterium violaceum. World J. Microbiol. Biotechnol. 42:52. doi: 10.1007/s11274-025-04715-x 41572086

[B100] Silva E. Teixeira J. A. Pereira M. O. Rocha C. M. R. Sousa A. M. (2023). Evolving biofilm inhibition and eradication in clinical settings through plant-based antibiofilm agents. Phytomedicine 119:154973. doi: 10.1016/j.phymed.2023.154973 37499434

[B101] Sionov R. V. Steinberg D. (2022). Targeting the holy triangle of quorum sensing, biofilm formation, and antibiotic resistance in pathogenic bacteria. Microorganisms 10:1239. doi: 10.3390/microorganisms10061239 35744757 PMC9228545

[B102] Slachmuylders L. Van Acker H. Brackman G. Sass A. Van Nieuwerburgh F. Coenye T. (2018). Elucidation of the mechanism behind the potentiating activity of baicalin against burkholderia cenocepacia biofilms. PLoS One 13:e190533. doi: 10.1371/journal.pone.0190533 PMC5749847 29293658

[B103] Song Y. Ye Z. Wang Y. (2026). Metabolite-driven reprogramming of bacterial persisters: mechanisms and therapeutic opportunities for overcoming antibiotic tolerance. Drug Resist. Updat. 84:101322. doi: 10.1016/j.drup.2025.101322 41202685

[B104] Sousa A. Phung A. N. Kalko-Basnet N. Obuobi S. (2023). Smart delivery systems for microbial biofilm therapy: dissecting design, drug release and toxicological features. J. Control. Release 354, 394–416. doi: 10.1016/j.jconrel.2023.01.003 36638844

[B105] Stenvang M. Dueholm M. S. Vad B. S. Seviour T. Zeng G. Geifman-Shochat S. . (2016). Epigallocatechin gallate remodels overexpressed functional amyloids in pseudomonas aeruginosa and increases biofilm susceptibility to antibiotic treatment. J. Biol. Chem. 291, 26540–26553. doi: 10.1074/jbc.M116.739953 27784787 PMC5159513

[B106] Stewart P. S. White B. Boegli L. Hamerly T. Williamson K. S. Franklin M. J. . (2019). Conceptual model of biofilm antibiotic tolerance that integrates phenomena of diffusion, metabolism, gene expression, and physiology. J. Bacteriol. 201, e307–e319. doi: 10.1128/JB.00307-19 PMC6805107 31501280

[B107] Su L. Li Y. Tian S. Huang F. Ren Y. Yang C. . (2022). Synergy between ph- and hypoxia-responsiveness in antibiotic-loaded micelles for eradicating mature, infectious biofilms. Acta Biomater. 154, 559–571. doi: 10.1016/j.actbio.2022.10.020 36243368

[B108] Suh K. Lee Y. Baek S. Kim J. Seo J. Yang Y. . (2025). Bakuchiol kills *Staphylococcus aureus* persisters and potentiates colistin activity against acinetobacter baumannii persisters. Front. Pharmacol. 16:1592183. doi: 10.3389/fphar.2025.1592183 40444041 PMC12119637

[B109] Sun T. Li X. Hong J. Liu C. Zhang X. Zheng J. . (2019). Inhibitory effect of two traditional chinese medicine monomers, berberine and matrine, on the quorum sensing system of antimicrobial-resistant *Escherichia coli*. Front. Microbiol. 10:2584. doi: 10.3389/fmicb.2019.02584 31798551 PMC6863804

[B110] Sun Y. Roy S. Yang Q. Tang Y. J. Ren D. (2025). Differential carbon source utilization drives metabolic state and resuscitation in antibiotic-tolerant persister cells. Front. Pharmacol. 16:1634627. doi: 10.3389/fphar.2025.1634627 40969939 PMC12440864

[B111] Svenningsen M. S. Veress A. Harms A. Mitarai N. Semsey S. (2019). Birth and resuscitation of (p)ppgpp induced antibiotic tolerant persister cells. Sci. Rep. 9:6056. doi: 10.1038/s41598-019-42403-7 30988388 PMC6465370

[B112] Tang H. Hao S. Khan M. F. Zhao L. Shi F. Li Y. . (2022). Epigallocatechin-3-gallate ameliorates acute lung damage by inhibiting quorum-sensing-related virulence factors of pseudomonas aeruginosa. Front. Microbiol. 13:874354. doi: 10.3389/fmicb.2022.874354 35547130 PMC9083413

[B113] Thurlow L. R. Hanke M. L. Fritz T. Angle A. Aldrich A. Williams S. H. . (2011). *Staphylococcus aureus* biofilms prevent macrophage phagocytosis and attenuate inflammation in vivo. J. Immunol. 186, 6585–6596. doi: 10.4049/jimmunol.1002794 21525381 PMC3110737

[B114] Van den Bergh B. Fauvart M. Michiels J. (2017). Formation, physiology, ecology, evolution and clinical importance of bacterial persisters. Fems Microbiol. Rev. 41, 219–251. doi: 10.1093/femsre/fux001 28333307

[B115] Virzì N. F. Greco V. Stracquadanio S. Jasim A. Greish K. Diaz-Rodriguez P. . (2024). Berberine-styrene-co-maleic acid nanomicelles: unlocking opportunities for the treatment and prevention of bacterial infections. RSC Adv. 14, 34066–34080. doi: 10.1039/D4RA04457F 39469023 PMC11513620

[B116] Wainwright J. Hobbs G. Nakouti I. (2021). Persister cells: formation, resuscitation and combative therapies. Arch. Microbiol. 203, 5899–5906. doi: 10.1007/s00203-021-02585-z 34739553 PMC8590677

[B117] Wang B. Wu B. Ma Y. Liu X. Tao L. Jia L. . (2025). Astragalus polysaccharides: structure-immunomodulation relationships, multi-target pharmacological activities, and cutting-edge applications in immune modulation. Front. Immunol. 16:1714898. doi: 10.3389/fimmu.2025.1714898 41383616 PMC12689378

[B118] Wang J. Jiao H. Meng J. Qiao M. Du H. He M. . (2019). Baicalin inhibits biofilm formation and the quorum-sensing system by regulating the msra drug efflux pump in staphylococcus saprophyticus. Front. Microbiol. 10:2800. doi: 10.3389/fmicb.2019.02800 31921008 PMC6915091

[B119] Wang J. Zhu J. Meng J. Qiu T. Wang W. Wang R. . (2021). Baicalin inhibits biofilm formation by influencing primary adhesion and aggregation phases in staphylococcus saprophyticus. Vet. Microbiol. 262:109242. doi: 10.1016/j.vetmic.2021.109242 34562786

[B120] Wang M. Fang K. Hong S. M. C. Kim I. Jang I. Hong S. H. (2018). Medium chain unsaturated fatty acid ethyl esters inhibit persister formation of *Escherichia coli* via antitoxin hipb. Appl. Microbiol. Biotechnol. 102, 8511–8524. doi: 10.1007/s00253-018-9271-3 30088019

[B121] Wang W. Feng X. Shi H. Wang Y. Jiang C. Xiao Z. . (2023). Biofilm inhibition based on controlling the transmembrane transport and extracellular accumulation of quorum sensing signals. Environ. Res. 221:115218. doi: 10.1016/j.envres.2023.115218 36608761

[B122] Wang Y. Zhang D. Sun Y. Zeng Y. Qi P. (2023). Precise localization and simultaneous bacterial eradication of biofilms based on nanocontainers with successive responsive property toward ph and atp. ACS Appl. Mater. Interfaces 15, 8424–8435. doi: 10.1021/acsami.2c22682 36744696

[B123] Watson M. Saitis T. Shareef R. Harb C. Lakhani M. Ahmad Z. (2023). Shikonin and alkannin inhibit atp synthase and impede the cell growth in *Escherichia coli. Int. J. Biol. Macromol*. 253(Pt 4):127049. doi: 10.1016/j.ijbiomac.2023.127049 37758110

[B124] Witzany C. Regoes R. R. Igler C. (2022). Assessing the relative importance of bacterial resistance, persistence and hyper-mutation for antibiotic treatment failure. Proc. R. Soc. B Biol. Sci. 289:20221300. doi: 10.1098/rspb.2022.1300 PMC9653239 36350213

[B125] Wu C. Lee S. Taylor C. Li J. Chan Y. Agarwal R. . (2020). Scientific and regulatory approach to botanical drug development: a u.s. Fda perspective. J. Nat. Prod. 83, 552–562. doi: 10.1021/acs.jnatprod.9b00949 31977211

[B126] Wu S. Yang K. Hong Y. Gong Y. Ni J. Yang N. . (2022). A new perspective on the antimicrobial mechanism of berberine hydrochloride against *Staphylococcus aureus* revealed by untargeted metabolomic studies. Front. Microbiol. 13:917414. doi: 10.3389/fmicb.2022.917414 35910599 PMC9328669

[B127] Wu S. Zhou M. Zhou H. Han L. Liu H. (2025). Astragaloside iv-loaded biomimetic nanoparticles target iκbα to regulate neutrophil extracellular trap formation for sepsis therapy. J. Nanobiotechnol. 23:155. doi: 10.1186/s12951-025-03260-x PMC11869569 40022068

[B128] Wu X. Pan B. Chu C. Zhang Y. Ma J. Xing Y. . (2025). Cxcl16/cxcr6/tgf–β feedback loop between m–mdscs and treg inhibits anti–bacterial immunity during biofilm infection. Adv. Sci. 12:2409537. doi: 10.1002/advs.202409537 PMC11831521 39716908

[B129] Xiao J. Yin M. Yang M. Ren J. Liu C. Lian J. . (2024). Lipase and ph-responsive diblock copolymers featuring fluorocarbon and carboxyl betaine for methicillin-resistant *Staphylococcus aureus* infections. J. Control. Release 369, 39–52. doi: 10.1016/j.jconrel.2024.03.021 38508523

[B130] Xing Y. Bian M. Huang X. Lv B. Huang Z. Jiang X. . (2025). Genome-wide screen reveals glycerol-induced aminoglycoside potentiation against *Staphylococcus aureus* via boosting glpk-initiated energy metabolism. Antimicrob. Agents. Chemother. 69, e925–e938. doi: 10.1128/aac.00938-25 PMC12691589 41128499

[B131] Xiong W. Lo J. Chou K. J. Wu C. Magnusson L. Dong T. . (2018). Isotope-assisted metabolite analysis sheds light on central carbon metabolism of a model cellulolytic bacterium clostridium thermocellum. Front. Microbiol. 9:1947. doi: 10.3389/fmicb.2018.01947 30190711 PMC6115520

[B132] Yadav V. Sahoo S. Malhotra N. Mishra R. Sreedharan S. Rajmani R. S. . (2025). Bioenergetic reprogramming of macrophages reduces drug tolerance in mycobacterium tuberculosis. Nat. Commun. 16:9370. doi: 10.1038/s41467-025-64407-w 41130947 PMC12549822

[B133] Yang D. Hao S. Zhao L. Shi F. Ye G. Zou Y. . (2021). Paeonol attenuates quorum-sensing regulated virulence and biofilm formation in pseudomonas aeruginosa. Front. Microbiol. 12:692474. doi: 10.3389/fmicb.2021.692474 34421847 PMC8371487

[B134] Yang T. Xie S. Cao L. Li M. Ding L. Wang L. . (2024). Astragaloside iv modulates gut macrophages m1/m2 polarization by reshaping gut microbiota and short chain fatty acids in sepsis. Shock 61, 120–131. doi: 10.1097/SHK.0000000000002262 37962207 PMC11841723

[B135] Yang X. Wang Y. Li L. Tang D. Yan Z. Li M. . (2025). Berberine and its nanoformulations and extracts: potential strategies and future perspectives against multi-drug resistant bacterial infections. Front. Microbiol. 16:1643409. doi: 10.3389/fmicb.2025.1643409 40964668 PMC12436466

[B136] Zafer M. M. Mohamed G. A. Ibrahim S. R. M. Ghosh S. Bornman C. Elfaky M. A. (2024). Biofilm-mediated infections by multidrug-resistant microbes: a comprehensive exploration and forward perspectives. Arch. Microbiol. 206:101. doi: 10.1007/s00203-023-03826-z 38353831 PMC10867068

[B137] Zhang Y. Ji W. Qin H. Chen Z. Zhou Y. Zhou Z. . (2025a). Astragalus polysaccharides alleviate dss-induced ulcerative colitis in mice by restoring scfa production and regulating th17/treg cell homeostasis in a microbiota-dependent manner. Carbohydr. Polym. 349(Pt A):122829. doi: 10.1016/j.carbpol.2024.122829 39643403

[B138] Zhang Y. Li N. Gong H. Zhao C. Bao X. Liu W. . (2025b). Structural characterization and anti-tumor immunomodulatory effects of polysaccharides from astragalus mongholicus with different cultivation modes. Int. J. Biol. Macromol. 318(Pt 4):145233. doi: 10.1016/j.ijbiomac.2025.145233 40516740

[B139] Zhang Y. Zhang Y. Ma R. Sun W. Ji Z. (2023). Antibacterial activity of epigallocatechin gallate (egcg) against shigella flexneri. Int. J. Environ. Res. Public Health 20:4676. doi: 10.3390/ijerph20064676 36981585 PMC10048926

[B140] Zhao L. Tan S. Zhang H. Liu P. Tan Y. Z. Li J. C. . (2018). Astragalus polysaccharides exerts anti–infective activity by inducing human cathelicidin antimicrobial peptidell−37in respiratory epithelial cells. Phytother. Res. 32, 1521–1529. doi: 10.1002/ptr.6080 29672953

[B141] Zheng J. Wang W. Gao X. Zhao S. Chen W. Li J. . (2022). Cascade catalytically released nitric oxide-driven nanomotor with enhanced penetration for antibiofilm. Small 18:e2205252. doi: 10.1002/smll.202205252 36344450

[B142] Zhou L. Liu Z. Wang Z. Yu S. Long T. Zhou X. . (2017). Astragalus polysaccharides exerts immunomodulatory effects via tlr4-mediated myd88-dependent signaling pathway in vitro and in vivo. Sci. Rep. 7:44822. doi: 10.1038/srep44822 28303957 PMC5355992

[B143] Zou Y. Zhang H. Zhang Y. Wu Y. Cheng J. Jia D. . (2023). A near-infrared light-triggered nano-domino system for efficient biofilm eradication: activation of dispersing and killing functions by generating nitric oxide and peroxynitrite via cascade reactions. Acta Biomater. 170, 389–400. doi: 10.1016/j.actbio.2023.08.038 37625678

